# CCL28 Enhances HSV-2 gB-Specific Th1-Polarized Immune Responses against Lethal Vaginal Challenge in Mice

**DOI:** 10.3390/vaccines10081291

**Published:** 2022-08-10

**Authors:** Yan Yan, Kai Hu, Ming Fu, Xu Deng, Xinmeng Guan, Sukun Luo, Mudan Zhang, Yalan Liu, Qinxue Hu

**Affiliations:** 1 Center of Clinical Laboratory, The Fifth People’s Hospital of Wuxi, Wuxi Affiliated Clinical Academy of Nantong University, Wuxi 214016, China; 2 State Key Laboratory of Virology, Wuhan Institute of Virology, Center for Biosafety Mega-Science, Chinese Academy of Sciences, Wuhan 430071, China; 3 Institute for Infection and Immunity, St. George’s University of London, London SW17 0RE, UK

**Keywords:** HSV-2, glycoprotein B, CCL28, Th1 immune response, viral challenge

## Abstract

Plasmid DNA (pDNA) represents a promising “genetic vaccine platform” capable of overcoming major histocompatibility complex barriers. We previously demonstrated that low-to-moderate doses of mucosae-associated epithelial chemokine (MEC or CCL28) as an immunomodulatory adjuvant can trigger effective and long-lasting systemic and mucosal HSV-2 gD-specific immune responses, whereas mice immunized with gD in combination with high-dose CCL28 showed toxicity and lost their immunoprotective effects after lethal HSV-2 challenge. The exact causes underlying high-dose, CCL28-induced lesions remain unknown. In an intramuscularly immunized mouse model, we investigated the immune-enhancement mechanisms of low-dose CCL28 as a molecular adjuvant combined with the relatively weak immunogen HSV-2 gB. Compared with the plasmid gB antigen group, we found that a low-dose of plasmid CCL28 (pCCL28) codelivered with pgB induced increased levels of gB-specific serum IgG and vaginal fluid IgA, serum neutralizing antibodies (NAb), Th1-polarized IgG2a, and cytokine IL-2 (>5-fold). Furthermore, low-dose pCCL28 codelivery with pgB enhanced CCL28/CCR10-axis responsive CCR10^−^ plus CCR10^+^ B-cell (~1.2-fold) and DC pools (~4-fold) in the spleen, CCR10^−^ plus CCR10^+^ T-cell pools (~2-fold) in mesenteric lymph nodes (MLNs), and the levels of IgA-ASCs in colorectal mucosal tissues, leading to an improved protective effect against a lethal dose of HSV-2 challenge. Findings in this study provide a basis for the development of CCL28-adjuvant vaccines against viral mucosal infections.

## 1. Introduction

Herpes simplex virus type 2 (HSV-2) infection is ubiquitous [1]. An estimated 491.5 million people worldwide were infected with HSV-2 in 2016, including 13.2% of the 15–49-year-old population [2]. HSV-2 is the primary pathogen of genital herpes, with symptomatic or asymptomatic infection during a lifelong latency [3]. Currently, the prevalence of HSV-2 varies depending on geographical area, age, gender, and race [1,2,4]. Another major public health significance of HSV-2 is its potential role in increasing HIV-1 susceptibility and transmission [4,5]. Given the magnitude of the HSV-2 epidemic and its role in HIV-1 infection, novel approaches are needed to tackle HSV-2 infection [6]. One of the most economical and effective ways to reduce the global burden of HSV-2 infection is to develop effective prophylactic HSV-2 vaccines [7]. However, despite tremendous efforts and expenditure over the last few decades, no licensed vaccine is available yet [6,8].

HSV-2 glycoprotein B (gB), like gD, a target antigen (Ag) for neutralizing antibodies (NAbs) and T cells, is frequently used in the development of HSV-2 vaccines [9]. Nevertheless, a subunit HSV-2 gB vaccine in combination with adjuvants consisting of 3-O-deacylated monophosphoryl lipid A and alum (MPL-alum), did not show adequate efficacy against HSV-2 infection in the population [10]. According to the latest clinical studies, an ideal genital herpes vaccine should not only prevent genital lesions and the infection of both HSV-1 and HSV-2 but also induce a long-lasting immune response that can last for decades [3]. It is known that compared to live-attenuated and inactivated viral vaccines, the major limitation of DNA vaccines is their low immunogenicity [9,11,12]. The molecular weight of HSV-2 gB is around two times higher than that of gD. We previously found that the Ag-specific Ab endpoint titer induced by plasmid gB (pgB) was significantly lower than that of plasmid gD [13,14]. Accordingly, in the absence of an adjuvant, following a lethal dose of HSV-2 challenge with HSV-2, pgB-immunized mice had higher disease severity scores, viral shedding incidence, and mortality rates compared to pgD-immunized mice. These findings support the lower immunogenicity of HSV-2 gB compared to gD. According to our previous conceptual vaccine studies [13,14], using a weak or lower dose of immunogen may be more appropriate if the intention is to investigate the effect of an adjuvant. Therefore, we chose gB as an immunogen to elucidate the molecular mechanism of CCL28 in enhancing host immune responses.

Chemokines can bridge innate and adapted immunity and/or guide the migration of immunocytes and have received wide attention as a novel molecular adjuvant [15,16]. The chemokine CCL28 is widely expressed in mucosal tissues, such as the mammary glands, salivary glands, intestines, and the trachea [17]. CCL28 has been studied as an adjuvant to improve mucosal immune response against HIV-1 [18,19,20,21] and influenza A [22]. CCL28 also has additional biological functions, such as broad-spectrum antimicrobial activities [23,24]. Given that CCL28 as a molecular adjuvant at a high dose demonstrated toxicities, as well as ineffective antiviral immunoprotective effects [13], in the current study, we investigated whether and how a low dose of plasmid CCL28 (pCCL28) can promote HSV-2 gB-induced protective immune responses. Findings of this study could facilitate the use of CCL28 in the development of effective HSV-2 prophylactic vaccines.

## 2. Materials and Methods

### 2.1. Plasmids

Murine CCL28 and HSV-2-truncated gB (730t) were subcloned into pcDNA3.1(+) vector (Invitrogen, the Netherlands) as described previously and designated as pCCL28 and pgB, respectively [14,20]. Eukaryotic expression of gB and CCL28 was confirmed both in vitro and in vivo [14,20]. Truncated gB (730t) was also subcloned into pET28a vector (Novagen, Shah Alam, Malaysia), designated pET-gB, for prokaryotic expression in an *Escherichia coli* system (Rosetta strain, Sangon Biotech, Shanghai, China) [14].

### 2.2. Animals, HSV-2, and Cell Lines

Female BALB/c mice aged six to eight weeks were purchased from Beijing HFK Biotechnology and fed under specific pathogen-free (SPF) conditions. HSV-2 (G strain) was propagated and titrated in Vero cells as described previously [14]. Vero and 293T cells were purchased from the American Type Culture Collection and cultured in Dulbecco’s Modified Eagle Medium (DMEM) containing 10% fetal bovine sera (FBS) (Invitrogen, Shanghai, China).

### 2.3. Prokaryotic Expression and Purification of gB

*Escherichia coli* Rosetta bacteria were subjected to pET-gB plasmid transformation and induced with isopropyl-β-D-thiogalactopyranoside (IPTG) to promote gB expression for 4 h. The bacterial cells were ultrasonically treated and centrifuged at 1200× *g* for 30 min at 4 °C, and the insoluble fractions were harvested. Insoluble gB protein was subsequently denatured in denaturing buffer over 14 h and refolded in refolding buffer for 24 to 48 h. The refolded gB solution was loaded onto a pre-equilibrated nickel-charged chelating Sepharose fast flow column (GE Healthcare). The six-His tag on the C terminus of the gB protein was used to facilitate purification, followed by elution with imidazole buffer. Subsequently, purified gB was dialyzed and concentrated. Protein concentration was determined using a BCA assay kit (Thermo Scientific Pierce, Shanghai, China). The protein molecular weight and the purity of prokaryotically expressed gB were assessed by SDS-PAGE and Western blot assays (*Monoclonal antibodies*: gB H126; Santa Cruz Biotechnology, Dallas, TX, USA) (Supplementary Materials Figure S1).

### 2.4. Mouse Immunization

**Figure 1:**
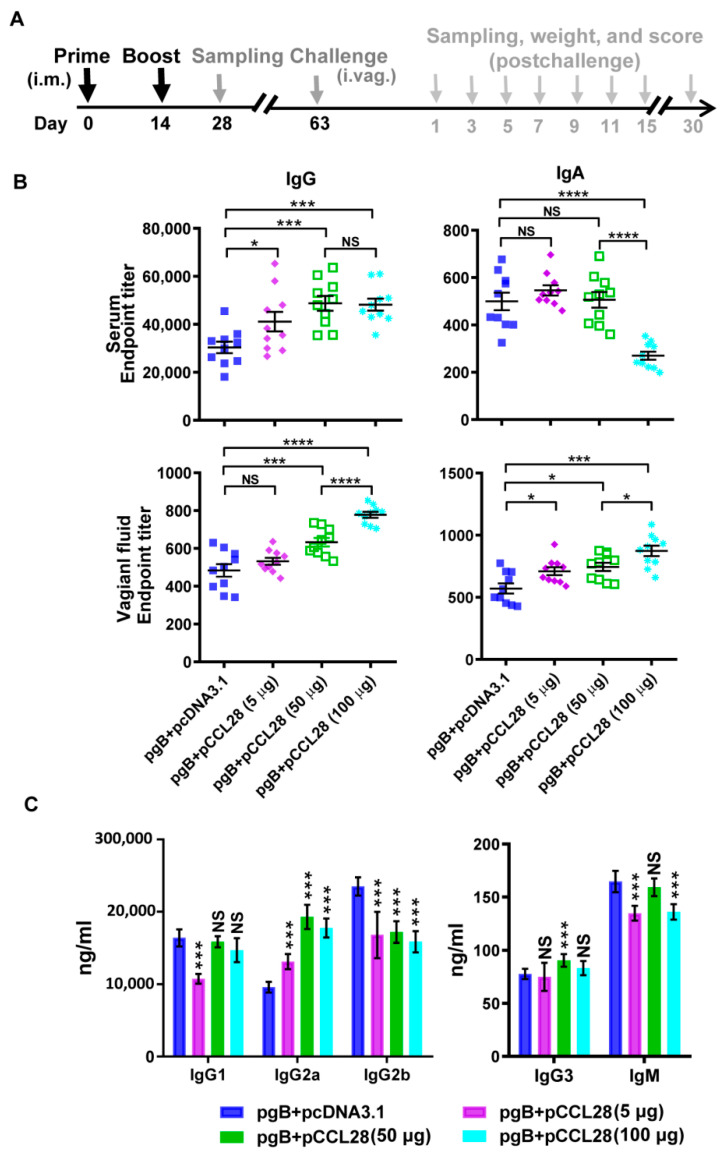
Immunization and challenge procedures, as well as humoral immune responses. (**A**) Schedule of i.m. immunization and i.vag. challenge. Mice (*n* = 10/group) were immunized twice with pcDNA3.1, pgB + pcDNA3.1, and pgB + pCCL28 (5, 50, and 100 μg, respectively) in saline solution on days 0 and 14. On day 14 post boost (day 28), some mice (*n* = 5/group) were sacrificed for sampling. Then, 49 days post boost (day 63), the remaining mice (*n* = 5/group) were used for challenge experiments. The weight and clinical symptoms of post-challenge mice were monitored daily for 15 days. Serum, vaginal fluid, and sacral ganglia samples were collected at the indicated time for subsequent tests. (**B**) Induction of systemic and mucosal Ab responses against gB. After mice underwent day 0 and 14 electroporation, as well as “prime and boost” i.m. immunization, the endpoint titers of gB-specific IgG and IgA of sera and vaginal fluids (*n* = 10/group) were determined by direct ELISA. (**C**) The concentrations of gB-specific IgG1, IgG2a, IgG2b, IgG3, and IgM isotypes in sera were quantified by ELISA post boost. On day 14 post boost, the titer of each gB-specific Ig subclass was calculated according to the standard curve obtained using the corresponding standard Ig subclass. Data are expressed as the means ± SEMs pooled from three independent experiments, with each condition performed in duplicate. The indicated *p*-values show statistically significant differences between the antigen group (pgB + pcDNA3.1) and the pgB + pCCL28 (5, 50 or 100 μg) group, as determined by a nonparametric Student’s *t*-test. NS, not statistically significant; * *p* < 0.05; *** *p* < 0.001; **** *p* < 0.0001.

According to our designed immunization procedures shown in Figure 1A, mice (*n* = 10 per group) were immunized twice two weeks apart using the “prime and boost” strategy, and an intramuscular (i.m.) immunization method was carried out as described previously [14]. For the test groups, pgB (5 μg) was mixed with low-to-high doses of pCCL28 (5, 50, or 100 μg) or a control vector (pcDNA3.1) to coimmunize the mice. For the negative control group, mice were immunized with pcDNA3.1 alone. In brief, plasmids were dissolved and mixed in sterile saline solution with a total volume of 40 μL per mouse, which were then delivered into the quadriceps muscle of one hind leg using electroporation. Shock conditions: 100 V, 50 ms, 3 shocks, and 3 more shocks post electrode reversal. A total of 2 doses of immunization were administered to each mouse over 14 days.

### 2.5. Murine Sera, Vaginal Fluids, and Tissue Sampling

On days 14 and 49 post boost, murine (*n* = 10/group) peripheral blood and vaginal fluid samples were taken from the fundus oculi and genital tract, respectively. Serum samples were obtained by centrifugation from the coagulated blood at 800× *g* for 15 min at room temperature. The vaginal fluid samples were collected by washing the vagina 3 times using PBS containing protease inhibitors (Roche, Basel, Switzerland) and collected in a total volume of 100 μL per mouse. Vaginal fluid samples were centrifuged at 12,000× *g* for 15 min at 4 °C, and the supernatants were harvested. These samples were aliquoted into small volumes and stored at −80 °C until use. Mice (*n* = 5 per group) were used to isolate the spleen and mesenteric lymph nodes (MLNs) under sterile conditions on day 14 post boost. Cell suspensions were obtained by pressing the tissues, followed by passing through Falcon^®^ 70 μm cell strainers (BD Biosciences, Shanghai, China). The red blood cells of single-cell suspensions were lysed using an erythrocyte lysis buffer (Sigma-Aldrich, Shanghai, China). Intratissue purified immunocytes were resuspended in RPMI 1640 medium and counted by an automated cell counter (Bio-Rad, Hercules, CA, USA).

### 2.6. Ag-Specific Immunoglobulin (Ig) and Ig Isotyping ELISA

Prokaryotic expression of HSV-2 gB protein was diluted with PBS to a concentration of 2.0 μg/mL and used for 96-well plate coating at 50 μL per well overnight at 4 °C. Briefly, the double-diluted samples collected on day 14 post boost were added to the plates and measured (the starting dilution ratio for sera was 1/5000, and that for vaginal fluids was 1/200) with endpoint ELISA assay. The values of the color intensity were read by an automated microplate ELISA reader (Tecan) at the wavelengths of *A450* and *A630* (for reference). The endpoint titers of gB-specific Igs were calculated using a combination of Excel and GraphPad Prism 8.0.2 software [14]. In the same dilution, murine samples read by OD_450_ were considered positive if they were higher than the OD_450_ of the negative control.

The concentration values of gB-specific isotype Igs in murine sera were measured by HRP-conjugated anti-mouse IgG1, IgG2a, IgG2b, IgG3, and IgM (the starting dilution ratio for sera was 1/5000; Southern Biotechnology Associates, Birmingham, AL, USA) according to the manufacturer’s instructions. The Ig isotype concentration was calculated according to the respective standard curve.

### 2.7. Virus Neutralization Assay

The neutralization abilities of murine sera and vaginal fluids were tested by a plaque assay according to our previous study [14]. Briefly, the murine antisera were heat-inactivated at 56 °C for 60 min. A volume of 150 μL serially diluted sera or vaginal fluids in DMEM (without FBS) was mixed and incubated with 150 μL HSV-2 (2.4 × 10^7^ PFU/mL) for 60 min at 37 °C and subsequently added to the Vero monolayer cells cultured in 96-well plates. The viral plaques were observed and recorded within 36–48 h. The neutralizing activities were determined based on the dilution titers that reduced the number of plaques compared with the positive control using the viral cytopathic effect (CPE) index.

### 2.8. Ag-Specific Cytokine Assay

Splenocytes (1 × 10^7^ per well, *n* = 5 mice per group) in 1 mL complete RPMI 1640 medium were added to 24-well plates. Purified gB protein (1 μg/mL) was added as a stimulant and cultured at 37 °C for 5 days. After incubation, filtered cell supernatants were used to detect the Th1/Th2 cytokines (IL-2, IL-4, IL-5, IFN-γ, and TNF-α) by mouse Th1/Th2 cytokine kits (BD Biosciences, Shanghai, China) according to the manufacturer’s instructions. The test and data acquisition were performed on the BD FACSAria III platform and analyzed by FCAP Array Software v3.0 (BD Biosciences, Shanghai, China).

### 2.9. Immunohistochemistry of Colorectal Tissues

On day 14 post boost, murine IgA^+^ cell numbers in colorectal tissues were analyzed according to the previously described experimental procedures [14]. Briefly, approximately 2 cm tissues from the end of the anus were cut and fixed in 10% methanol, stained by the primary Ab of goat anti-mouse IgA Ab (AbD Serotec, Oxford, UK) at 4 °C, labeled, and color-developed by the secondary Ab using a two-step immunohistochemical kit (GBI, Shanghai, China) according to the manufacturer’s instructions. The IgA^+^ cells were stained brown by 3,3′-diaminobiphenyl biphenyl-free base (DBA) reagent and subsequently counterstained with hematoxylin.

### 2.10. Chemotaxis Assay

On day 14 post boost, freshly isolated 2 × 10^6^ cells/300 μL of splenocytes or mesenteric lymph node lymphocytes (MLNLs) were added into the upper chambers of 3.0 μm pore-size Transwell 12-well plates (Corning Costar, Corning, NY, USA). A volume of 600 μL complete RPMI-1640 medium with (testing well) or without (control well) recombinant murine CCL28 (500 ng/mL, R&D Systems, Minneapolis, MN, USA) was added to the lower chambers in duplicate for each sample. The plates were incubated in a 37 °C, 5% CO_2_ incubator for 2 h. Finally, the upper chambers were discarded. The migrated cells in the lower chambers were pipetted, mixed, and counted. Fold changes of migrated cells = the average number of migrated cells in testing wells/the average number of migrated cells in control wells.

### 2.11. Cell Surface Staining and Analysis of Murine Lymphocytes

Fresh isolated 2 × 10^6^ cells/mouse of splenocytes or MLNLs for each group were analyzed by flow cytometry. Separated cells were dyed with rabbit anti-mouse CD16/CD32, CD3e-PE, CD19-PE-Cy7, and CD11c-APC (BD Biosciences, Shanghai, China), as well as goat anti-mouse CCR10-Axlexa Flour 488 (Abcam, Cambridge, UK), at 4 °C for 30 min. The stained cells were washed and detected on a BD FACSAria III platform. The absolute number of CD3e^+^, CD19^+^, or CD11c^+^ cells (1 × 10^5^/mouse) from each immunization group and control group (pcDNA3.1) was analyzed by flow cytometry. CCR10 expression on CD3e^+^, CD19^+^, or CD11c^+^ cells was analyzed using FlowJo V10 software (BD Biosciences, Shanghai, China).

### 2.12. Challenge, Scoring, and Virus Quantification

Five to seven days before the challenge, 2 mg of Depo-Provera (medroxyprogesterone acetate) was injected under the neck ruffs of immunized mice. On day 49 post boost, mice (*n* = 5 per group) were anesthetized with pentobarbital sodium and injected intravaginally (i.vag) with 10 μL HSV-2 (2.4 × 10^7^ PFU/mL) per mouse. Sera were collected for virus neutralization assay on days 5, 9, and 11 post challenge, whereas vaginal fluid samples were collected on days 1, 3, 5, 7, 9, 11, and 15 post challenge for detection of virus shedding. Infected mice were monitored daily for weight loss, vaginal inflammation scores, and death according to the 0–5 scoring criteria by Toka et al. [25]: 0, no apparent infection; 1, mild inflammation of the external genitals, redness, and moderate swelling of external genitals; 3, severe redness and inflammation; 4, genital ulceration and severe inflammation; 5, hind limb paralysis and death. On day 30 post challenge or the day of animal death, the murine sacral ganglia were collected to detect the latent viral DNA load with quantitative real-time PCR (qPCR) assays. The copies of the HSV-2 genomes were quantified in triplicate using probe qPCR with a premix Ex Taq^TM^ kit (TaKaRa, Dalian, China) according to the manufacturer’s instructions on a Roch LightCycler^®^ 480. The following reference primers were used in amplification reactions [26]: 5′gG-356 (5′- GCC TGC CGT CAG CCC ATC CTC CT -3′) and 3′gG-508 (5′- TCG GCA CCA GCA GGG AAG CAT TT -3′), as well as the probe 5′- (FAM) CCT TCG GCA GTA TGG AGG GTG TCG C (TAMRA) -3′.

### 2.13. Statistical Analysis

Statistical analyses were performed using GraphPad Prism 8.0.2 (Version 7.0, San Diego, CA, USA). The data are presented as the means ± standard error of the means (SEMs). The comparisons between the two groups were analyzed by Student’s *t*-test, and the comparisons among more than three groups were analyzed by one-way ANOVA analysis or the Kruskal–Wallis nonparametric analysis test. *p* < 0.05 was considered statistically significant.

## 3. Results

### 3.1. Codelivery of CCL28 with gB Plasmids Enhances gB-Specific, Th1-Polarized Humoral Responses

Mice were immunized twice with pgB in combination with pcDNA3.1 (pgB + pcDNA3.1) or pCCL28 (pgB + pCCL28) or immunized with pcDNA3.1 as the negative control on days 0 (prime) and 14 (boost) to examine whether codelivery with pCCL28 could enhance gB-specific humoral immune responses (Figure 1A). On day 14 post boost, we first compared the levels of increased immunogenicity induced by coadministration of pgB with different doses of pCCL28. Sera and vaginal fluids were collected to compare the production of gB-specific IgG and IgA. In comparison with the pgB + pcDNA3.1 group, mice immunized with pgB + pCCL28 (5, 50, or 100 μg) produced significantly high levels of gB-specific serum IgG and vaginal IgA. In contrast, codelivery of pgB with a high dose of pCCL28 (HDCCL28) induced a significantly low level of serum IgA (Figure 1B). These data suggest that, unlike HDCCL28, low-to-moderate doses of pCCL28 (LMDCCL28) as an adjuvant may play a critical role in enhancing pgB-induced humoral immune responses.

Previous research has shown that mice immunized with plasmids encoding HSV-2 gD or gB produced high levels of Ag-specific IgG subclasses in sera, which almost exclusively contained IgG1 rather than IgG2a or IgG2b [27,28,29]. It is known that IgG1 is associated with a Th2-polarized immune response, whereas other IgG subclasses are associated with a Th1-polarized immune response, with the IgG2a/IgG1 ratio being the most important indicator for Th1- or Th2- polarized immune response [13]. To examine the impact of CCL28 on the isotypes of Ab responses, we measured serum Ig isotypes, including IgG1, IgG2a, IgG2b, IgG3, and IgM, on day 14 post boost. As shown in Figure 1C, coadministration of LMDCCL28 with pgB induced a higher IgG2a/IgG1 ratio (Th1-polarized) compared to the pgB + pcDNA3.1 group. Dose-dependent effects on IgG1, IgG2a, IgG3, and IgM levels were observed between 5 and 50 μg (or 100 μg on IgG1 and IgG2a levels) CCL28-adjuvanted groups. These findings indicate that LMDCCL28 can increase the induction of HSV-2 gB-specific, Th1-like Ab responses.

### 3.2. Codelivery of a Low Dose of CCL28 (LDCCL28) with gB Plasmids Promotes Enhanced Viral Neutralization Activities and Protective Effects Post Challenge

**Figure 2:**
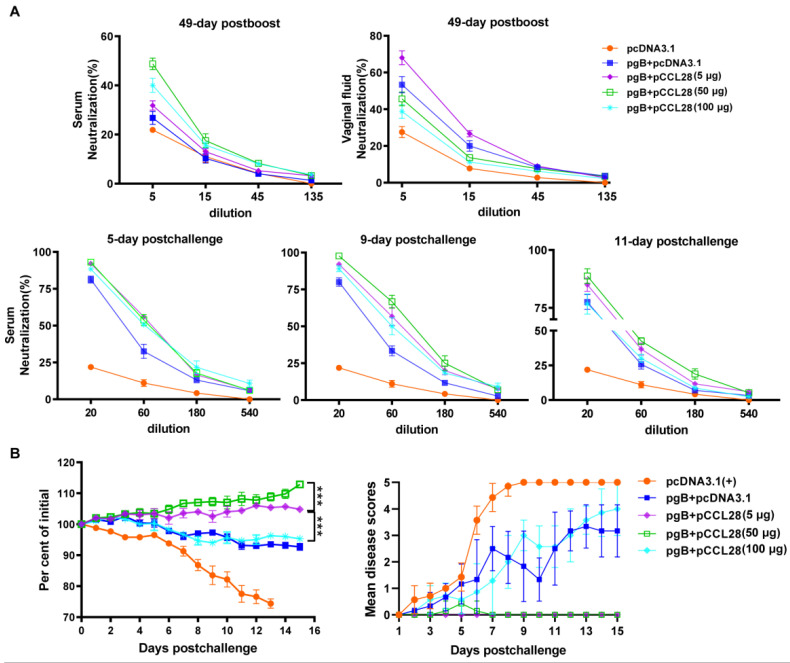
Neutralizing activities of murine sera and vaginal fluids and the protective effects on immunized mice against vaginal HSV-2 challenge. (**A**) Neutralizing activities of murine sera and vaginal fluids. Sera of immunized mice (*n* = 5/group) were collected on the indicated days, and the neutralizing activities were tested at a starting dilution of 1:5 (on day 49 post boost) or 1:20 (on days 5, 9, and 11 post challenge). Vaginal fluids of immunized mice (*n* = 5/group) were collected on day 49 post boost, and the neutralizing activities were tested at a starting dilution of 1:5. The CPE index was evaluated within 36–48 h, and the percentages of the inhibition curves were plotted. (**B**) Weight loss (**left panel**) and disease severity (**right panel**) were monitored up to 15 days post challenge. Data are expressed as the means ± SEMs pooled from at least three independent experiments, with each condition performed in duplicate. *** *p* < 0.001; pgB + pCCL28 (5 μg) compared to pgB + pCCL28 (50 or 100 μg) group by Kruskal–Wallis nonparametric analysis of variance (ANOVA).

An effective prophylactic HSV-2 vaccine has been shown to induce powerful and long-lasting NAb responses after immunization and virus challenge [12,13,30]. We investigated whether codelivery of CCL28 with gB plasmids can functionally modulate viral neutralization activities. The serum-neutralizing activities were tested on day 49 post boost (before challenge) and on days 5, 9, and 11 post challenge, whereas vaginal fluid-neutralizing activities were tested on day 49 post boost. As shown in Figure 2A, the neutralizing activities of sera and vaginal fluids against HSV-2 were significantly increased in mice immunized with pgB + pCCL28 (5 μg) on day 49 post boost, and the serum-neutralizing activities were significantly increased on days 5, 9, and 11 post challenge compared with the pgB + pcDNA3.1 (antigen) group. The NAbs at the lowest dilution (1:5) from sera and vaginal fluids in the MHDCCL28 groups were significantly lower than those in the LDCCL28 group on day 49 post boost. These results indicate that LDCCL28 may be sufficient to elicit long-lasting NAb responses post immunization. Furthermore, after the lethal vaginal HSV-2 challenge, the NAbs of mice immunized with pgB + pCCL28 (5 μg) were recalled on day 5, peaked on day 9, and declined on day 11 compared with the antigen group. These data suggest that LDCCL28 can help to enhance systemic and mucosal NAb responses, that such enhancement is sustainable, and that the neutralizing activities can be reactivated immediately post challenge.

After being challenged with HSV-2 on day 49 post boost, the mice’s weight and clinical symptoms were monitored daily for up to 15 days. Mice in the control group developed significant weight loss, and the loss became more obvious after day 5. Mice immunized with pgB + pcDNA3.1 or with pgB + pCCL28 (100 μg) developed slight weight loss. In contrast, mice immunized with pgB + pCCL28 (5 or 50 μg) showed a significant weight increase post challenge compared with the antigen group (Figure 2B, left panel). Unlike the other groups, mice immunized with pcDNA3.1 developed severe disease symptoms until death, and all mice died on day 13 post challenge. Nearly 40% of mice immunized with pgB + pcDNA3.1 or pgB + pCCL28 (100 μg) developed severe but relatively mild disease symptoms (inflammation, genital ulceration, and hair loss) compared to the pcDNA3.1 group. In contrast to those with pCCL28 (50 μg), mice coimmunized with pgB + pCCL28 (5 μg) did not develop significant disease symptoms post challenge (Figure 2B, right panel). Furthermore, 100 μg pCCL28 induced ruffled hair and immune protection insufficiency or loss. Taken together, as shown in Figure 2A, 49 days post boost mice immunized with moderate-to-high doses of pCCL28 (MHDCCL28) exhibited high neutralizing activities, whereas the neutralizing activities of vaginal fluids were lower compared with those from the LDCCL28 group. As shown in Figure 2B (right panel), serum-neutralizing activities induced by MHDCCL28 seemed less effective in protecting against HSV-2, suggesting the need to induce a stronger neutralizing activity in the genital tract. Given that LDCCL28 was sufficient to promote gB to produce immune responses against HSV-2 challenge, gB + pCCL28 (5 μg) was chosen for the subsequent studies. 

### 3.3. LDCCL28 Coimmunized with gB Plasmids Induce Ag-Specific Th1-Polarized Cellular Immune Responses

**Figure 3:**
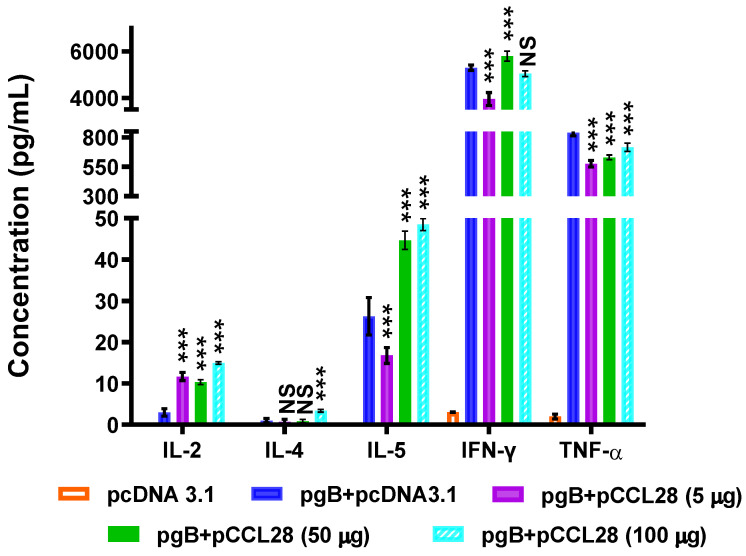
gB-specific Th1/Th2-related cytokine production. Comparison of cytokines produced by splenocytes in each group of mice (*n* = 5/group). On day 14 post boost, splenocytes (1 × 10^7^ cells) isolated from each immunized group were stimulated with purified gB (1 μg/mL) and cultured for 5 days, and subsequently, the supernatants were collected for cytokine assessment using a CBA kit. The productions of Th1- (IL-2, IFN-γ, TNF-α) and Th2 (IL-4, IL-5)-related cytokines are shown as the means of the cytokine concentrations (pg/mL) ± SEMs pooled from at least two independent experiments, with each condition performed in duplicate. The indicated *p*-values show statistically significant differences between the antigen group and the pgB + pCCL28 (5, 50 or 100 μg) group, as determined by a nonparametric Student’s *t*-test. NS, not statistically significant; *** *p* < 0.001.

CCL28 has been identified as a key regulator of Ag-specific T-cell- and DC-dependent adaptive immune responses, lymphoid tissue hyperplasia responses, and indirect B-cell upregulation in vivo [20]. Herein, we hypothesize that codelivery of LDCCL28 with the gB plasmid can mediate an Ag-specific Th1/Th2-like cellular immune response and thus improve the protective effects against HSV-2, likely by secreting cytokines that may mediate the regulation of the composition of resident immunocytes. Given the importance of cytokines secreted by activated immunocytes in defining the types of immune responses, we measured Ag-specific Th1- (IL-2, TNF-α, IFN-γ) and Th2 (IL-4, IL-5)-related cytokine profiles to assess cellular immune polarization. Compared with the antigen group, unlike the pgB + pCCL28 (50 or 100 μg) group, mice immunized with pgB + pCCL28 (5 μg) produced significantly high levels of IL-2 (>5-fold) and low levels of IL-5, IFN-γ, and TNF-α. Murine splenocytes in the pgB + pCCL28 (50 or 100 μg) group produced significantly high levels of IL-5 (Figure 3). These data highlight that LDCCL28-induced, IL-2-dominatented, Th1-polarized cellular responses may play an important role in immunoprotection.

### 3.4. Coimmunization of LDCCL28 with gB Plasmids Enhances Immunocyte Migration and Settlement in Secondary Lymph Sites

**Figure 4:**
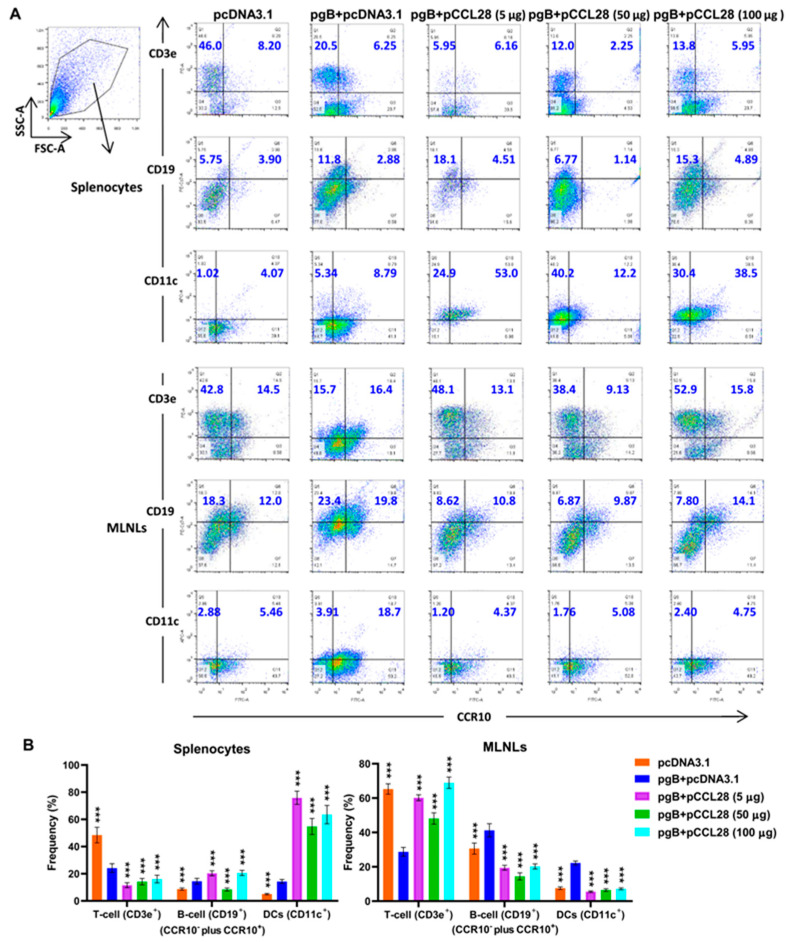
Comparison of gB-specific CCR10^+/−^ immunocyte compositions in the spleen and mesenteric lymph nodes. (**A**) Flow cytofluorometry graphs of splenocytes and mesenteric lymph node lymphocytes (MLNLs). On day 14 post boost, splenocytes and MLNLs (1 × 10^7^ cells) isolated from various groups (*n* = 5/group) were stimulated with purified gB (1 μg/mL), and the frequencies (in percentage) of CCR10^+/−^ CD3e^+^, CD19^+^, and CD11c^+^ immunocytes were detected by Flow cytometry assay and analyzed by FlowJo software. (**B**) Comparison of the frequencies (%) of murine CCR10^−^ plus CCR10^+^ immunocytes in each group. Data are expressed as the means of immunocyte frequencies (%) ± SEMs pooled from at least two independent experiments, with each condition performed in duplicate. Statistically significant differences were determined by comparing with the pgB + pcDNA3.1 group. *** *p* < 0.001; antigen group compared to control (pcDNA3.1) or pgB + pCCL28 (5, 50, or 100 μg) group, as determined by nonparametric Student’s *t*-test.

CCL28 binding to the CCR3 or CCR10 receptor plays an important role in building a bridge between innate and adaptive immunity by recruiting leukocytes to the secondary lymphoid tissues [13,17]. Expression of receptor CCR3 or CCR10 is elevated in the inflammatory process [31,32] and T-cell lymphoma [33]. Having demonstrated that LDCCL28 enhanced the gB-specific systemic and mucosal Ab responses, as well as Th1-related cytokine production, and that there were no serious disease manifestations after virus challenge, we further investigated the roles of LDCCL28 in directing the migration of immunocytes to the secondary immune organs and tissues. Given that spleen and MLNs are the important secondary immune organ or tissues in mice and involved in lymphocyte homing and recycling [34], we used a flow cytometry assay to examine the expression of CCR10^−/+^ immunocytes (T cells, B cells, and DCs) in the spleen and MLNs. In the spleen, LDCCL28 significantly promoted not only the frequencies of CCR10^−/+^ CD19^+^ B cells but also the frequencies of CCR10^−/+^ CD11c^+^ DCs compared with the antigen group. In contrast, LDCCL28-induced CCR10^−^ CD11c^+^ DCs and CCR10^+^ CD11c^+^ DCs frequencies were up to ~5-fold and ~6-fold higher compared with those of the antigen group, respectively. Compared to the MHDCCL28 group, LDCCL28 induced lower frequencies of CCR10^−^ CD11c^+^ DCs and higher frequencies of CCR10^+^ CD11c^+^ DCs. In MLNLs, LDCCL28 only significantly promoted the frequencies of CCR10^−^ CD3e^+^ T-cells by ~3-fold, whereas the frequencies of CCR10^+^ CD3e^+^ T-cells, CCR10^−/+^ CD19^+^ B-cells, and CCR10^−/+^ CD11c^+^ DCs in each CCL28 group were reduced compared with those from the antigen group (Figure 4A). As shown in Figure 4B, mice immunized with pgB + pCCL28 (5 μg) produced amplified pools of CCR10^+^ plus CCR10^−^ CD19^+^ cells (~1.2-fold) and CD11c^+^ cells (~4-fold) in the spleen, as well as CCR10^−^ plus CCR10^+^ CD3e^+^ cells in MLNs (~2-fold) compared with the pgB + pcDNA3.1 group. The T-cell frequencies in the spleen and MLNs significantly decreased after pgB + pcDNA3.1 immunization (blue bar) compared with the control group (orange bar). In the spleen, LDCCL28 (pink bar) decreased the induction of gB-associated T-cell frequencies but enhanced gB-associated B-cell and DC frequencies. In MLNLs, LDCCL28 enhanced gB-associated T-cell frequencies but decreased gB-associated B-cell and DC frequencies compared with the antigen group. Therefore, LDCCL28 might increase the migration and settlement of responsive CCR10^−/+^ B cells and DCs in the spleen and CCR10^−^CD3e^+^ T cells in MLNLs on day 14 post boost, which could help to enhance the protective immune responses against HSV-2 challenge.

### 3.5. Codelivery of LDCCL28 and gB Plasmids Enhances the Responsive Immunocytes Chemoattracting to Colorectal Mucosal and Secondary Lymphoid Sites

**Figure 5:**
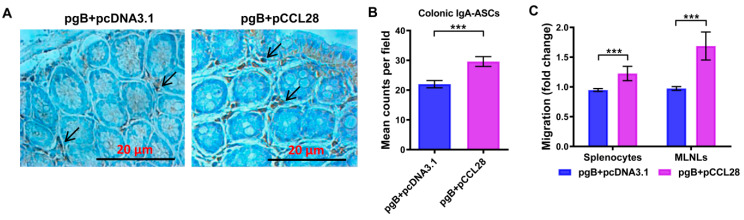
Recruitment of immunocytes mediated by the CCL28 adjuvant in tissues. (**A**) Comparison of IgA^+^ cells at the colorectal mucosal sites of immunized mice (magnification: ×200). Murine colorectal samples (*n* = 5/group) were collected on day 14 post boost, and IgA^+^ cells were detected by immunohistochemistry. The colorimetric reaction was developed with diaminobenzidine and counterstained with H&E. The arrows indicate IgA^+^ cells. (**B**) Quantification of IgA^+^ cells by counting five high-power fields for each colorectal sample. Data are expressed as the means of IgA^+^ cells ± SEMs for each group. (**C**) Chemotactic splenocytes and MLNLs of immunized mice in response to murine CCL28 protein. On day 14 post boost, the splenocytes and MLNLs (1 × 10^7^/mL) were prepared and counted and used to assess the chemotactic activities recruited by CCL28 protein using a Transwell system. The migrated cells in the lower chamber were counted after 2 h incubation. Fold changes were calculated by comparing with the number of cells in the lower chamber without CCL28 protein. Data are expressed the means of migrated cell fold changes ± SEMs for each group. *** *p* < 0.001, antigen group compared to pgB + pCCL28 (5 μg) group, as determined by nonparametric Student’s *t*-test.

CCL28 is one of the chemokines that can recruit leukocytes to secondary lymphoid tissues and IgA antibody-secreting cells (IgA-ASCs) to mucosal surfaces at mucosal sites [13,17]. HSV-2 is primarily transmitted through mucosal sites of the rectum or genital tract, and IgA is considered to be a primary adaptive immune response against pathogens at mucosal sites. In our study, a significant increase in vaginal Abs was observed in mice immunized with pgB + pCCL28 (5 μg). We therefore assessed the number of rectal IgA^+^ cells. Colorectal immunohistochemistry analyses revealed that mice immunized with pgB + pCCL28 (5 μg) developed a significant increase in IgA^+^ cells compared with the antigen group (Figure 5A,B). These results suggest that LDCCL28 could exert an ability to attract more IgA^+^ cells to migrate to the rectal mucosa sites, which might play an important role in immune protection during HSV-2 challenge.

Freshly isolated splenocytes and MLNLs were used to evaluate the chemotactic abilities induced by murine CCL28 protein in vitro in order to evaluate whether LDCCL28 as an adjuvant can effectively enhance the chemotactic abilities of the immunocytes in the secondary lymphoid organs or tissues. As shown in Figure 5C, mice immunized with pgB + pCCL28 (5 μg) produced a significantly high level of responsive immunocytes to the spleen and MLNs compared with the antigen group, but the enhancements in the spleen were lower than those in MLNs. In general, codelivery of LDCCL28 with gB plasmids can result in significantly higher levels of IgA^+^ plasma cells at colorectal mucosal sites and more immunocytes migrating to mucosal immunity-related MLNs post boost.

### 3.6. Codelivery of LDCCL28 and gB Plasmids Reduces Viral Shedding and Latency Post Lethal Vaginal Challenge

The frequencies and loads of virus shedding in the reproductive tract reveal the natural course and pathogenesis of HSV-2 infection. One approach to vaccine development is to reduce the amount of virus that establishes latency in neurons and the amount of virus shedding from genital surfaces [12]. Previously, several vaccine candidates showed the potential to reduce the amount of HSV-2 in the sacral nerves of mice or guinea pigs [26]. To determine whether codelivery of LDCCL28 with gB plasmids can efficiently establish an immune defense against a lethal dose of vaginal HSV-2 challenge, 7 weeks post boost, we sampled vaginal fluids and sacral ganglia of the i.vag.-challenged mice at the indicated time points (Figure 1A), and virus shedding and latent viral DNA load were subsequently assessed.

**Figure 6:**
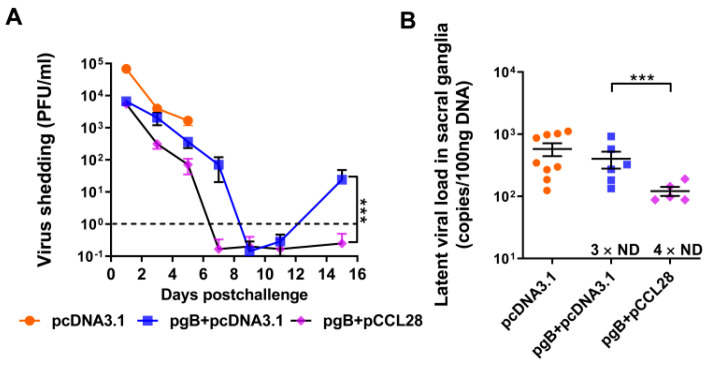
Virus shedding and latent viral DNA of immunized mice post challenge. On day 49 post boost, mice (*n* = 5/group) were challenged i.vag. with a lethal dose of HSV-2 (2.4 × 10^7^ PFU/mL). (**A**) Virus shedding in the vaginal fluids was determined by plaque assay at the indicated time points. The dashed line indicates the detection limit of the virus. (**B**) Latent viral DNA loads. The viral DNA in sacral ganglia samples was quantified by quantitative real-time PCR (qPCR) on day 30 post challenge or the day of death. Data are expressed as the means ± SEMs pooled from at least three independent experiments, with each condition performed in duplicate. Number × ND, means the number of mice in which latent virus cannot be detected; *** *p* < 0.001; antigen group compared to pgB + pCCL28 (5 μg) group, as determined by nonparametric Student’s *t*-test.

As shown in Figure 6A, viral loads in vaginal fluid samples were detectable but dropped sharply daily in all groups. The negative control group (pcDNA3.1) developed the highest virus-shedding rate, whereas the pgB + pcDNA3.1 group developed moderate viral release. The pgB + pCCL28 (5 μg) group developed the lowest viral load, decreasing sharply. The mice in the pcDNA3.1 group died starting on day 7, and all died by day 13 post challenge; due to severe disease manifestations, their vaginal fluid samples were only collected up to day 6. As shown in Figure 6A, mice coinjected with LDCCL28 survived, and the viral loads in vaginal fluid samples dropped to below the detection limit between days 7 and 9. Notably, virus reshedding was observed in the pgB + pcDNA3.1 group on day 15 post challenge (Figure 6A). Table 1 summarizes the incidences of HSV-2 shedding in murine vaginal fluids, showing a similar tendency. From day 7 to 15 post challenge, no viral DNA was detected in the pgB + pCCL28 (5 μg) group.

qPCR was used to quantify the copies of latent HSV-2 in the sacral ganglia samples of mice on day 30 post challenge or at the time of death. The HSV-2 genome copies, from high to low, ranked in order of pcDNA3.1, pgB + pcDNA3.1, and pgB + pCCL28 (5 μg) groups, implying that LDCCL28 coimmunization with gB could prevent HSV-2 from establishing latent infection post challenge (Figure 6B). We investigated the dose effect of CCL28 on HSV-2 gB immunogenicity (positive effects on IgG titers; Figure 1B) and the protective efficacies against a lethal dose of HSV-2 challenge (reverse effects on mean disease scores; Figure 2B, right panel). The results revealed that immunization with LDCCL28 in combination with gB can protect mice against HSV-2 challenge, whereas HDCCL28 appeared to induce side effects.

Overall, we found that in combination with gB, LDCCL28 as an adjuvant boosted humoral and cellular responses and improved viral clearance, likely mediated by a Th1-polarized cellular response. Our findings indicate that the adjuvant effect of CCL28 is probable due to its capability of promoting the migration of responsive immunocytes and the induction of Th1-polarized cellular immunity, thereby providing mucosal immune protection against HSV-2 infection.

## 4. Discussion

**Figure 7:**
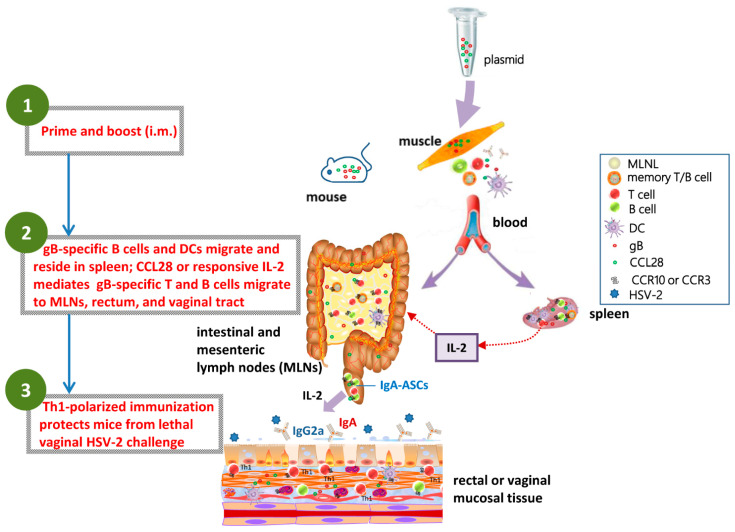
Diagram of how CCL28 mediates gB-specific, Th1-polarized immune responses to protect against vaginal challenge in mice. DNA vaccines containing pgB + pCCL28 can distribute and express throughout the body via blood flow after i.m. injection. When peripheral blood immunocytes circulate, they follow the bloodstream to the spleen, MLNs, and rectal sites, whereas CCR10^+^ B cells and CCR10^+^ DCs tend to reside in the spleen, CCR10^+^ T cells tend to reside in MLNs, and CCL28-responsive IgA-ASCs tend to reside in rectal sites. The diagram shows how CCL28 enhances IL-2-initiated, Th1-polarized immunity and promotes the immune responses to effectively protect mice against HSV-2 challenge.

An alternative prophylactic HSV-2 vaccine should induce potent humoral, cellular, and mucosal immunity to sterilize HSV-2 shedding and to reduce virus recurrence in the long term [9]. HSV-2 gB has a larger molecular mass than gD, and the adjuvant effects of CCL28 on gB have yet to be elucidated. Given that a high dose of pCCL28 in combination with HSV-2 pgD [13] induced significant side effects post i.m. immunization, here, we used HSV-2 gB as the immunogen to investigate the adjuvant effects of CCL28 and the underlying mechanisms. In the current study, we found that LDCCL28, when used as a molecular adjuvant, can effectively boost gB immunogenicity, which protected mice from lethal vaginal challenge and latency. Mechanistically, LDCCL28 effectively enhanced Ag-specific immune responses, including mucosal Ab responses and Th1-polarized cellular responses, and such enhanced responses can be quickly recalled. The adjuvant effects of enhancing Ag-specific immune responses appeared to be associated with the amplification of CCL28/CCR10 axis-responsive immunocyte pools, as reflected by the migration of CCR10^−/+^ immunocytes and IgA-ASCs from distant organs into mucosal and secondary lymphoid sites (Figure 7).

The primary target of vaccines against mucosally acquired viral infections is the induction of broad and potent NAbs at mucosal sites. Clinical trial studies have revealed that the tested HSV-2 vaccine candidates had difficulty inducing optimal Ab levels, which are required to prevent viral invasion [3]. Given that chemokines have been shown to have a fairly broad range of adjuvant effects [15,25,35], we initially used CCL28 in combination with HSV-2 gB to determine whether protective efficacies could be achieved post challenge. We found that in combination with HSV-2 gB, CCL28 had a reverse dose-dependent effect in protecting mice against HSV-2 challenge. The data shown in Figure 2B and Figure 6, and Table 1 were generated from the same animals. Although mice immunized with 50 μg CCL28 plasmid had similar weight changes and disease scores compared to the LDCCL28 group, HSV-2 shedding in the MHDCCL28 group resulted in significantly higher viral PFU values in vaginal fluid samples and latent viral copies in sacral ganglia than those in the LDCCL28 group post challenge. Given that MHDCCL28 induced apparent side effects, the corresponding results are not listed in Figure 6 and Table 1. Inconsistent with our previous report that less CCL28 than CCL19 was required to achieve a similar adjuvant effect [13], codelivery of LDCCL28 and HSV-2 gB plasmids significantly increased IgG and IgA levels in sera post boost. We also engineered fusion constructs pCCL28IZgB and pgBIZCCL28 using IZ linker, as described previously [13]; however, immunization with these two constructs did not result in protective immunity in mice (data not shown). Although it remains to be determined in a future study, CCL28 fused with gB likely affected the trimeric conformation or epitope exposure of gB and consequently failed to elicit ideal gB-specific immune responses. Previously, a guinea pig study revealed that gB-specific IgG2a contributes to a potent neutralization capacity against HSV-2 challenge [30]. Our present study showed that LDCCL28 in combination with gB enhanced the level of IgG2a (Th1-polarized) production. Furthermore, whereas mice immunized with pgB + pcDNA3.1 displayed a rapid decline in the level of serum-neutralization Abs, mice coimmunized with pgB + pCCL28 (5 μg) developed sustained and rapid recalled immune response following HSV-2 challenge. These findings suggest that LDCCL28-induced, Th1-polarized humoral immune responses likely play an important role in immunoprotection.

We also found that CCL28 as an adjuvant enhanced gB-specific, Th1-polarized cellular immune responses, such as an increased IL-2 cytokine secretion by splenocytes, which could play a critical role in preventing viral invasion. We found that murine splenocytes in the LDCCL28-coimmunized group produced IL-2 dominantly instead of IFN-γ and TNF-α compared with the antigen group. IL-2 can be used as a growth factor for T cells in vitro and as a necessary cytokine for the induction of effective immune responses in vivo. IL-2 also has a pluripotent role, such as mediating the activation of leukocyte-activated killer cells and the functions of macrophages and B cells [36]. Although IL-2 can be secreted by T, B, and dendritic cells (DCs) in peripheral tissues, and T cells and DCs in the thymus, how IL-2 facilitates immune-protective processes remain elusive [36]. In addition, IL-2R signaling is required to develop regulatory T-cell (Treg) biology and can regulate Treg homeostasis and inhibition [37]. Therefore, CCL28 as an adjuvant or immunotherapeutic agent is likely inextricably linked to the biological role played by IL-2. Furthermore, we found that MHDCCL28 induced significantly high levels of IL-5. IL-5 is mainly secreted by activated Th2 cells, mast cells, NK cells, NKT cells, and eosinophils; is involved in the differentiation, proliferation, migration, activation, and survival of eosinophils; and is associated with increased eosinophils and asthma severity [38]. It is known that CCL28 and its receptors are directly associated with organ damage [24,32]. The severe side effects observed in this study, such as ruffled hair and loss of immunoprotective roles, could be associated with MHDCCL28-induced, Th2-polarized IL5 production.

Chemokines can be secreted by mature dendritic cells (mDCs), and the chemokine/receptor axis plays a direct role in lymphocyte homing and pathogen encounters [13]. The binding of a chemokine to its cognate receptors can result in receptor internalization, which plays a role in regulating chemokine activities [39]. As the adjuvant effect of pCCL28 has been reported in our previous studies [20], here, we focused on investigating the dose-dependent effects of pCCL28 in combination with HSV-2 pgB in inducing humoral immune responses. We further assessed the protection efficacies against a lethal dose of virus challenge by evaluating mouse weight changes and disease scores. By comparing with the pgB + pcDNA3.1 group, we found that the proportions of CCR10^−^ CD3e^+^ T cells in MLNLs, CCR10^−/+^ CD19^+^ B cells, and CCR10^−/+^ CD11c^+^ DCs in the spleens of the pgB + pCCL28 (5 μg) group were significantly increased, likely in association with the host’s cellular immune activation after chemokine binding [13,40]. We also found that the pgB + pcDNA3.1 group was differed significantly from all other groups in terms of enhancing T-cell frequency in MLNLs. We speculate that HSV-2 gB may be involved in impairing T-cell immune responses. Therefore, more mechanistic studies are needed for functional verification. Another limitation of the present study is that we did not test whether CCR3^+/−^ cells exhibited similar patterns of change in mice. According to cellular frequency analyses of immune tissues, T cells, rather than the other two types of immunocytes, increased in MLNLs of the pgB + pCCL28 (5 μg) group (Figure 7), suggesting that T cells likely completed proliferation after pgB + pCCL28 (5 μg) immunization. Furthermore, the effects of recruitment or chemotaxis of IgA^+^ plasma cells to mucosal sites, as well as immunocyte homing or recycling to mucosal sites, are critical in immune surveillance. According to previous studies [13,20], we chose the more accessible rectal tissue instead of vaginal mucosal tissue. We speculate that the LDCCL28-promoted cellular immune responses likely played an essential role in enhancing NAbs and T-cell-mediated immune responses, protecting mice against HSV-2 infection. These findings collectively highlight that LDCCL28 has the potential to be used as an adjuvant in the development of prophylactic vaccines.

## 5. Conclusions

Our study showed that coimmunization of LDCCL28 with gB plasmids induced enhanced systemic and mucosal responses against HSV-2 infection. In addition to NAbs, an IL-2-related, Th1-polarized immune response could be useful as an indicator in evaluating effective vaccines against mucosally transmitted pathogens. To the best of our knowledge, this is the first study exploring the functions of CCL28 as a molecular adjuvant to boost HSV-2 gB-specific immune responses in an animal challenge model. Several studies have suggested that CCL28 is likely associated with inflammation and tumorigenesis, so its application prospects are called into question. Although studies have been conducted on CCL28 as a molecular adjuvant, the mechanisms of its potential side effects in immunization remain to be further addressed. Using HSV-2 gB as an immunogen, we confirmed the double-edged sword effects of CCL28 as an adjuvant and further explored its potential in vaccination. The relevant protective effects of CCL28 in combination with HSV-2 gB are associated with enhanced Th1-polarized immune responses and the recruitment of CCL28-responsive immunocytes to distant tissues. 

## Figures and Tables

**Table 1:** Incidences of virus shedding in mice post HSV-2 challenge (%) *.

Group	Total Animals	No. (%) of Mice Challenged with HSV-2 and Virus-Shedding Incidences						
		Day 1	Day 3	Day 5	Day 7	Day 9	Day 11	Day 15
**pcDNA3.1**	15	15 (100.0)	15 (100.0)	15 (100.0)	−	−	−	−
**pgB + pcDNA3.1**	15	15 (100.0)	15 (100.0)	12 (80.0)	9 (60.0)	ND	ND	3 (20.0)
**pgB + pCCL28**	15	15 (100.0)	9 (60.0)	5 (33.3)	ND	ND	ND	ND

*, HSV-2 shedding incidences in vaginal fluid samples (*n* = 5/group) post challenge were detected by qPCR. Data were pooled from at least three independent experiments. HSV-2 shedding incidence = (the number of vaginal fluids in which viral DNA was detected/ the total number of vaginal fluids analyzed) × 100. −, not obtainable for serious disease or death. The dose of pCCL28 was 5 μg. ND, no virus was detected.

## Data Availability

Data are included in the article and are also available on request from the corresponding author.

## Supplementary Materials

The following supporting information can be downloaded at: https://www.mdpi.com/article/10.3390/vaccines10081291/s1, Figure S1: Prokaryotic expression of gB protein verified by Western blot assay.

## Abbreviations

gB, glycoprotein B; CCL28, chemokine (C-C motif) ligand 28; DC, dendritic cell; IgA-ASC, IgA Ab-secreting cell; i.m., intramuscular(ly); i.vag., intravaginal(ly); MLN, mesenteric lymph node; MLNL, mesenteric lymph node lymphocyte; p, plasmid; PBS-T, PBS plus 0.05% Tween-20; PFU, plaque formation unit.
