# Supplementary material for: CCL28 Enhances HSV-2 gB-Specific Th1-Polarized Immune Responses against Lethal Vaginal Challenge in Mice
Source: Vaccines (Basel). 2022 Aug 10;10(8):1291. doi: 10.3390/vaccines10081291 (PMC9415327; doi:10.3390/vaccines10081291)
Supplement: Supplementary file 1 [file vaccines-10-01291-s001.zip › vaccines-1827953-SI.pdf]

Supplentary material

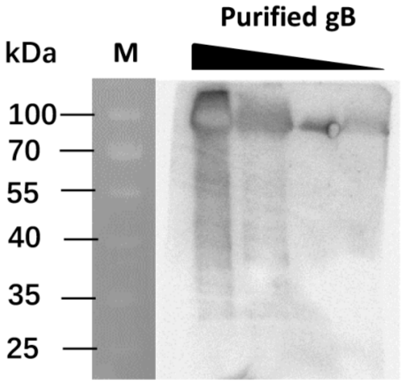

Supp. Figure S1. Prokaryotic expression of gB protein verified by Western blot assay. The purity of gB was verified by Western blot assay. Purfied gB was doble diluted after dialysis and detected with SDS-PAGE and Western blot assays. M: Protein ladder.
